# A hybrid continuous-discrete method for stochastic reaction–diffusion processes

**DOI:** 10.1098/rsos.160485

**Published:** 2016-09-14

**Authors:** Wing-Cheong Lo, Likun Zheng, Qing Nie

**Affiliations:** 1 Department of Mathematics, City University of Hong Kong, Kowloon, Hong Kong; 2 Department of Mathematics, University of California, Irvine, CA, USA; 3 Center for Mathematical and Computational Biology, University of California, Irvine, CA, USA

**Keywords:** reaction–diffusion systems, stochastic simulation, hybrid method, biological morphogen systems

## Abstract

Stochastic fluctuations in reaction–diffusion processes often have substantial effect on spatial and temporal dynamics of signal transductions in complex biological systems. One popular approach for simulating these processes is to divide the system into small spatial compartments assuming that molecules react only within the same compartment and jump between adjacent compartments driven by the diffusion. While the approach is convenient in terms of its implementation, its computational cost may become prohibitive when diffusive jumps occur significantly more frequently than reactions, as in the case of rapid diffusion. Here, we present a hybrid continuous-discrete method in which diffusion is simulated using continuous approximation while reactions are based on the Gillespie algorithm. Specifically, the diffusive jumps are approximated as continuous Gaussian random vectors with time-dependent means and covariances, allowing use of a large time step, even for rapid diffusion. By considering the correlation among diffusive jumps, the approximation is accurate for the second moment of the diffusion process. In addition, a criterion is obtained for identifying the region in which such diffusion approximation is required to enable adaptive calculations for better accuracy. Applications to a linear diffusion system and two nonlinear systems of morphogens demonstrate the effectiveness and benefits of the new hybrid method.

## 1. Introduction

Many biological systems are subject to stochastic fluctuations when the copy number of the molecules is relatively small [1,2]. For spatially inhomogeneous systems that involve both reactions and diffusions, the spatial distributions of the molecules are important because local copy number fluctuations may result in phenotypic differences, though the total number of molecules of the relevant species may be high [3].

For a spatially homogeneous system, the Gillespie stochastic simulation algorithm (SSA) tracks each reaction event and updates the state of the system after each occurrence of reactions [4]. Numerous methods have been developed to improve the efficiency of this method for cases involving large sizes for certain species or frequent reactions; examples of such methods include the next reaction method [5], *τ*-leaping [6–8], the hybrid methods for simulating fast and slow reactions [9–11] and the adaptive multi-level simulation algorithm [12].

For a spatially inhomogeneous system, the SSA can be applied by first partitioning the spatial domain into many compartments. In each compartment, reactions are treated as in the homogeneous case; however, molecules may jump between adjacent compartments through diffusion [13]. In this approach, the size of each compartment must be sufficiently small that diffusive jumps occur more rapidly than reactions and the inhomogeneity inside each compartment can be ignored [14,15]. Also, the time scale for the molecule to diffuse throughout a compartment should be much faster than the time scale for the fastest bimolecular chemical reaction [14,16,17].

In the case of frequent diffusive jumps, the spatial SSA may become computationally inefficient. Consequently, modifications have been made to accelerate the SSA, as in the next sub-volume method [18] and the null process [19], or to develop a new computational algorithm that optimizes the search process in the SSA [20], or to approximate diffusion, as in the multinomial simulation algorithm [21], the diffusive finite state projection method [22] and adaptive algorithms [23,24]. These approximation methods share a common feature: reaction and diffusion processes are simulated independently, and the diffusive jumps occur at predetermined time intervals, between which the reactions are simulated. As a result, the time step depends on the fastest diffusion rate, leading to excessive computational cost, in particular, for the case of fast diffusion.

Several hybrid methods were developed for solving stochastic reaction–diffusion systems [25–28]. The most common approach is a spatially hybrid method between the SSA and deterministic approach. Noise effect in high concentration region is relativity small so deterministic approach such as using partial differential equations is sufficient to ensure the accuracy of simulations. However, small noise effect in high concentration region may still result in large fluctuations in low concentration region because of communication between the regions, so the development of a hybrid method which combines two or more stochastic approaches without involving any deterministic approach becomes an important and effective approach.

Here, we introduce a hybrid continuous-discrete method to simulate spatially inhomogeneous systems with better efficiency and accuracy. As in the Central Limit Theorem, a continuous Gaussian random variable can be used to approximate the change in the number of molecules introduced by a large number of independent diffusive jumps. A brief overview of the algorithm is listed below:

1. Calculating the time of the next occurring reaction based on the SSA.
2. Approximating the numbers of diffusive jumps using Gaussian random vectors with time-dependent means and covariances by assuming no reactions during the period of time.
3. If the approximated number of diffusive jumps at some compartments is large enough, the approximation is then applied at that locations; otherwise the SSA is applied. Since the number of diffusive jumps may be different at each time period between reactions, so the locations at which the approximation is applied are set adaptively over time.

As a result, the updating takes place only when a reaction occurs, and the time step is then determined by the reaction rates. Because the correlation among diffusive jumps is considered in our method, the approximation of the diffusion is reliable, and its effect on the reactions is small.

The method is applied to 3 one-dimensional reaction–diffusion systems for morphogen gradients in addition to a two-dimensional morphogen system. The efficiency of the method improves as the diffusion coefficients increase or the number of diffusing molecules increases, yielding more than 60% savings in computational cost compared with the standard spatial SSA. In addition, because of the new time-adaptive criterion for identifying the region for the diffusion approximation, our hybrid method allows for zero initial conditions (i.e. an initial state without any molecules) in the simulations, unlike many other existing stochastic methods, which need to start with a certain level of molecules in order to obtain good approximations.

## 2. Material and methods

### 2.1. Chemical master equations for spatially inhomogeneous systems

Consider a system along a one-dimensional domain with length *L*, where *N* molecular species {*S*_1_,*S*_2_,…,*S*_*N*_} are involved in the following *M* reactions {*R*_1_,*R*_2_,…,*R*_M_}:

$$R_{j}:s_{j1}^{r}S_{1}+\cdots +s_{jN}^{r}S_{N}\overset{\gamma_{j}}{\to }s_{j1}^{p}S_{1}+\cdots +s_{jN}^{p}S_{N}.$$

Here, $s_{ji}^{r}$ and $s_{ji}^{p}$ are the stoichoimetric coefficients of the reactant and product species, respectively, and *γ*_*j*_ is the macroscopic rate constant of *R*_*j*_. Although we describe the method on a one-dimensional domain, it is worth noting that it is easy to extend this method in a higher dimensional domain. The examples in one- and two-dimensional domains will be discussed in the Simulation results section.

If the system is spatially inhomogeneous, then the domain is partitioned into *K* identical compartments with uniform length *h*, where *h*=*L*/*K*. The subsystem in each compartment is assumed to be homogeneous. Molecules in different compartments are treated as different species, denoted by {*S*_11_,*S*_12_,…,*S*_*ki*_,…,*S*_*KN*_}, where *S*_*ki*_ is the *i*th species in the *k*th compartment. The system state is denoted by

$$X(t)=(X_{11}(t),X_{12}(t),\ldots ,X_{ki}(t),\ldots ,X_{KN}(t)),$$

where *X*_*ki*_ is the number of molecules of *S*_*ki*_.

Only molecules in the same compartment can react. The *j*th reaction in the *k*th compartment *R*_*kj*_ is as follows:

$$R_{kj}:s_{j1}^{r}S_{k1}+\cdots +s_{jN}^{r}S_{kN}\overset{\gamma_{kj}}{\to }s_{j1}^{p}S_{k1}+\cdots +s_{jN}^{p}S_{kN},$$

where *γ*_*kj*_ is the reaction rate constant of reaction *R*_*kj*_.

Diffusion is treated as a reaction in which a molecule in one compartment jumps to one of its neighbouring compartments at a constant rate. Without loss of generality, we assume that only species *S*_1_ diffuses in the algorithm description. Assume that species *S*_1_ diffuses with the coefficient *D*_1_ with reflective boundary conditions on the boundary of the domain. Then, the diffusive jumps obey the following chain reactions:

$$S_{11}\overset{D_{1}/h^{2}}{\underset{D_{1}/h^{2}}{⇌}}S_{21}\overset{D_{1}/h^{2}}{\underset{D_{1}/h^{2}}{⇌}}S_{31}\cdots \overset{D_{1}/h^{2}}{\underset{D_{1}/h^{2}}{⇌}}S_{N1}.$$

Multiple diffusive species can be easily treated using the same approach, and an example of such a case is presented in the Simulation results section.

Consider *X*(*t*) as a variable *x*, the probability that the reaction *R*_*kj*_ will fire in the next time interval [*t*,*t*+d*t*) is *a*_*kj*_(*x*) d*t*, where *a*_*kj*_ is called the propensity function of *R*_*kj*_. The state of the system transfers from one state to another through reaction firing. The net change of the state of the system caused by one occurrence of reaction *R*_*kj*_ is denoted as *ν*_*kj*_ and so

$$\nu_{kj}=(0,\ldots ,0,\underset{\mathrm{from}\;((k-1)N+1)\mathrm{th}\;\mathrm{to}\;kN\mathrm{th}}{\underbrace{s_{j1}^{p}-s_{j1}^{r},\ldots ,s_{jN}^{p}-s_{jN}^{r}}},0,\ldots ,0).$$

In addition, denote as *a*_*kL*_(*x*) and *a*_*kR*_(*x*) the propensity functions of diffusion jumps $J_{kL}:S_{k1}\to S_{(k-1)1}$ and $J_{kR}:S_{k1}\to S_{(k+1)1}$, respectively. Denote as *ν*_*kL*_ and *ν*_*kR*_ the net change of the state of the system caused by *J*_*kR*_ and *J*_*kL*_, respectively. As diffusion is treated as mono-molecular reactions, we have

$$a_{kL}(x)=\frac{D_{1}}{h^{2}}X_{k1},\;\text{for }2<k\leq K,\;\text{and}\;a_{kR}(x)=\frac{D_{1}}{h^{2}}X_{k1},\;\text{for }1\leq k<K-1$$

and the elements of *ν*_*iL*_ and *ν*_*iR*_ equal 1, −1 or 0.

The chemical master equation (CME) that governs the temporal evolution of the probability density function *p*(*x*,*t*) that the state of the system is *x* at time *t* is as follows:

2.1 $$\begin{array}{rl} \frac{\partial }{\partial t}p(x,t) & =\underset{\mathrm{Left}\;\mathrm{jump}}{\underbrace{-\sum_{k=2}^{K}a_{kL}(x)p(x,t)+\sum_{k=2}^{K}a_{kL}(x-\nu_{kL})p(x-\nu_{kL},t)}}\\ & \;\underset{\mathrm{Right}\;\mathrm{jump}}{\underbrace{-\sum_{k=1}^{K-1}a_{kR}(x)p(x,t)+\sum_{k=1}^{K-1}a_{kR}(x-\nu_{kR})p(x-\nu_{kR},t)}}\\ & \;\underset{\mathrm{Reaction}}{\underbrace{-\sum_{k=1}^{K}\sum_{j=1}^{M}a_{kj}(x)p(x,t)+\sum_{k=1}^{K}\sum_{j=1}^{M}a_{kj}(x-\nu_{kj})p(x-\nu_{kj},t)}}. \end{array}$$

### 2.2. Diffusion approximation

For most biological systems, the state space and the dimension of the CME are large or infinite, rendering the CME impossible to solve. The stochastic process underlying the CME can be simulated by the spatial SSA (details can be found in appendix A). Similar to the existing methods [14–17], the compartment size is first appropriately chosen such that diffusive jumps are usually more frequent than reactions because of the assumption of the homogeneity of reactions in each compartment. In the case of frequent diffusive jumps, the spatial SSA may become computationally inefficient. Here we shall approximate the diffusion processes using Gaussian random vectors with time-dependent means and covariances.

Given *X*(*t*_0_)=*x*_0_=(*x*_0_11__,*x*_0_12__,…,*x*_0_*ki*__,…,*x*_0_*KN*__), let *n*_*kL*_(*t*) and *n*_*kR*_(*t*) denote the numbers of leftward and rightward diffusive jumps of molecules from the *k*th compartment between time *t*_0_ and time *t*. Under the assumption that a large number of diffusive jumps occurs between reactions in each compartment, we can approximate the numbers of diffusive jumps as follows:

2.2 $$\frac{n_{kL}(t)}{\Lambda }=\phi_{kL}(t)+\frac{\xi_{kL}}{\sqrt{\Lambda }}+O\left(\frac{1}{\Lambda }\right)$$

and

2.3 $$\frac{n_{(k-1)R}(t)}{\Lambda }=\phi_{(k-1)R}(t)+\frac{\xi_{(k-1)R}}{\sqrt{\Lambda }}+O\left(\frac{1}{\Lambda }\right),$$

for *k*=2,3,…,*K*. The functions *ϕ*_*kL*_(*t*) and *ϕ*_*kR*_(*t*) characterize the macroscopic features of the diffusive jumps. When the copy number of molecules is large enough, the system of the average of molecule concentration will approach a deterministic diffusion system. The macroscopic features of the diffusive jumps are determined by the solution of the deterministic system. The second terms *ξ*_*kL*_ and *ξ*_*kR*_ are real random numbers, measuring the fluctuations around *ϕ*_*kL*_(*t*) and *ϕ*_*kR*_(*t*). The means of these random numbers are zero and the covariances can be determined by the formula presented later in this section. The constant *Λ* is the number of molecules per unit concentration in a compartment. For example, if the concentration 1 μM corresponds to 600 molecules in each compartment, then *Λ*=600 μM^−1^. When *Λ*≫1, *ϕ*_*kL*_(*t*) and *ϕ*_*kR*_(*t*) are considered to be continuous functions of *t*.

Let *Π*(*ξ*_1*L*_,*ξ*_2*L*_,*ξ*_2*R*_,…,*ξ*_*nL*_,*t*) be the probability density function of *ξ*_1*L*_, *ξ*_2*L*_, *ξ*_2*R*_,…,*ξ*_*nL*_ at time *t*. The function *Π* for molecule concentrations can be obtained from a rescaling of the probability density function *p*, which is for the number of molecules. The formula for *Π* is as follows:

$$\Pi (\xi_{1L},\xi_{2L},\xi_{2R},\ldots ,\xi_{nL},t)=\frac{1}{\Lambda^{2n-2}}p\left(x_{0}+\sum_{k=1}^{K-1}n_{kR}\nu_{kR}+\sum_{k=2}^{K}n_{kL}\nu_{kL},t|x_{0},t_{0}\right),$$

where *p*(*x*,*t* | *x*_0_,*t*_0_) is the probability density function that the state of the system is *x* at time *t* with the condition *X*(*t*_0_)=*x*_0_.

By performing the standard systematic expansion of the master equation equation (2.1) [29], we obtain the following equation:

$$\begin{array}{rl} & \frac{\partial \Pi }{\partial t}-\sqrt{\Lambda }\left(\frac{\partial \Pi }{\partial \xi_{1R}},\frac{\partial \Pi }{\partial \xi_{2L}},\ldots ,\frac{\partial \Pi }{\partial \xi_{KL}}\right)\cdot \left(\frac{\partial \phi_{1R}(t)}{\partial t},\frac{\partial \phi_{2L}(t)}{\partial t},\ldots ,\frac{\partial \phi_{KL}(t)}{\partial t}\right)\\ & \;=\sqrt{\Lambda }[-\frac{D_{1}}{h^{2}}(c_{0_{11}}-\phi_{1R}+\phi_{2L})\frac{\partial \Pi }{\partial \xi_{1R}}-\frac{D_{1}}{h^{2}}\left(c_{0_{K1}}-\phi_{KL}+\phi_{(K-1)R}\right)\frac{\partial \Pi }{\partial \xi_{KL}}\\ & \;-\frac{D_{1}}{h^{2}}\sum_{k=2}^{K-1}(c_{0_{k1}}-\phi_{kR}-\phi_{kL}+\phi_{(k+1)L}+\phi_{(k-1)R})\frac{\partial \Pi }{\partial \xi_{kR}}\\ & \;-\frac{D_{1}}{h^{2}}\sum_{k=2}^{K-1}(c_{0_{k1}}-\phi_{kR}-\phi_{kL}+\phi_{(k+1)L}+\phi_{(k-1)R})\frac{\partial \Pi }{\partial \xi_{kL}}]\\ & \;+\frac{D_{1}}{h^{2}}\sum_{k=1}^{K-1}\frac{\partial }{\partial \xi_{kR}}(\xi_{kR}\Pi -\xi_{(k+1)L}\Pi )+\frac{D_{1}}{h^{2}}\sum_{k=2}^{K-1}\frac{\partial }{\partial \xi_{kR}}(\xi_{kL}\Pi -\xi_{(k-1)R}\Pi )\\ & \;+\frac{D_{1}}{h^{2}}\sum_{k=2}^{K}\frac{\partial }{\partial \xi_{kL}}\left(\xi_{kL}\Pi -\xi_{(k-1)R}\Pi \right)+\frac{D_{1}}{h^{2}}\sum_{k=2}^{K-1}\frac{\partial }{\partial \xi_{kL}}(\xi_{kR}\Pi -\xi_{(k+1)L}\Pi )\\ & \;+\frac{D_{1}}{2h^{2}}\frac{\partial^{2}}{\partial \xi_{1R}^{2}}\left(\left(c_{0_{11}}-\phi_{1R}+\phi_{2L}\right)\Pi \right)+\frac{D_{1}}{2h^{2}}\frac{\partial^{2}}{\partial \xi_{KL}^{2}}((c_{0_{K1}}-\phi_{KL}+\phi_{(K-1)R})\Pi )\\ & \;+\frac{D_{1}}{2h^{2}}\sum_{k=2}^{K-1}\frac{\partial^{2}}{\partial \xi_{kR}^{2}}((c_{0_{k1}}-\phi_{kR}-\phi_{kL}+\phi_{(k+1)L}+\phi_{(k-1)R})\Pi )\\ & \;+\frac{D_{1}}{2h^{2}}\sum_{k=2}^{K-1}\frac{\partial^{2}}{\partial \xi_{kL}^{2}}((c_{0_{k1}}-\phi_{kR}-\phi_{kL}+\phi_{(k+1)L}+\phi_{(k-1)R})\Pi )\\ & \;-\sum_{l=1}^{K}\sum_{j=1}^{M}a_{lj}\left(\Lambda \left(c_{0}+\sum_{k=1}^{K-1}\phi_{kR}\nu_{kR}+\sum_{k=2}^{K}\phi_{kL}\nu_{kL}\right)\right)\Pi +O\left(\frac{1}{\sqrt{\Lambda }}\right), \end{array}$$

where *c*_0_=(*c*_0_11__,*c*_0_12__,…,*c*_0_*KN*__)=*x*_0_/*Λ*. Assuming

2.4 $$\Lambda \gg 1\;\text{and}\;\max_{1\leq j\leq M,1\leq k\leq K}a_{kj}\ll \frac{D_{1}}{h^{2}}\min_{1\leq k\leq K-1}\left\{\frac{\partial \Pi }{\partial \xi_{kR}},\frac{\partial \Pi }{\partial \xi_{(k+1)L}}\right\},$$

and comparing the order term $\sqrt{\Lambda }$, we have

$$\begin{array}{rl} & \frac{\partial }{\partial t}\left(\begin{array}{c} \phi_{1R}\\ \phi_{2L}\\ \phi_{2R}\\ \vdots \\ \phi_{KL} \end{array}\right)=\frac{D_{1}}{h^{2}}\left(\begin{array}{c} c_{0_{11}}-\phi_{1R}+\phi_{2L}\\ c_{0_{21}}-\phi_{2R}-\phi_{2L}+\phi_{3L}+\phi_{1R}\\ c_{0_{21}}-\phi_{2R}-\phi_{2L}+\phi_{3L}+\phi_{1R}\\ \vdots \\ c_{0_{K1}}-\phi_{KL}+\phi_{(K-1)R} \end{array}\right),\\ & {\left(\phi_{1R},\phi_{2L},\phi_{2R},\ldots ,\phi_{KL}\right)}^{\prime }=0\;\text{at }t=t_{0}. \end{array}$$

Letting

2.5 $$\Pi_{s}=\Pi \exp\left(-\int_{t_{0}}^{t}\sum_{l=1}^{K}\sum_{j=1}^{M}a_{lj}\left(\Lambda \left(c_{0}+\sum_{k=1}^{K-1}\phi_{kR}(\tau )\nu_{kR}+\sum_{k=2}^{K}\phi_{kL}(\tau )\nu_{kL}\right)\right)d\tau \right)$$

and defining the vector variable (*y*_1_,*y*_2_,*y*_3_,…,*y*_2*K*−2_)′=(*ξ*_1*R*_,*ξ*_2*L*_,*ξ*_2*R*_,…,*ξ*_*KR*_)′, we obtain the following Fokker–Planck equation:

$$\frac{\partial \Pi_{s}}{\partial t}=-\sum_{i,j}A_{ij}\frac{\partial }{\partial y_{i}}(y_{j}\Pi_{s})+\frac{1}{2}\sum_{i,j}B_{ij}(t)\frac{\partial^{2}\Pi_{s}}{\partial y_{i}\partial y_{j}}+O\left(\frac{1}{\Lambda }\right),$$

where

$$A=\frac{D_{1}}{h^{2}}\left(\begin{array}{cccccccc} -1 & 1 & \\ 1 & -1 & -1 & 1 & \\ 1 & -1 & -1 & 1 & \\ & & 1 & -1 & -1 & 1 & \\ & & 1 & -1 & -1 & 1 & & \\ & & & \ddots & \ddots & \ddots & \ddots & \\ & & & & 1 & -1 & -1 & 1\\ & & & & 1 & -1 & -1 & 1\\ & & & & & & 1 & -1 \end{array}\right)$$

and

$$B(t)=\frac{D_{1}}{h^{2}}\left(\begin{array}{ccccc} c_{0_{11}}-\phi_{1R}+\phi_{2L} & & & & \\ & c_{0_{21}}-\phi_{2R}-\phi_{2L}+\phi_{1R}+\phi_{3L} & & & \\ & & c_{0_{21}}-\phi_{2R}-\phi_{2L}+\phi_{1R}+\phi_{3L} & & \\ & & & \ddots & \\ & & & & c_{0_{K1}}-\phi_{KL}+\phi_{(K-1)R} \end{array}\right).$$

So the corresponding covariance matrix is

$$\rho =\int_{0}^{t}Y(t)Y^{-1}(\tau )B(\tau ){\left(Y^{-1}(\tau )\right)}^{\prime }Y{\left(t\right)}^{\prime }\;d\tau ,$$

where Y(t)=exp⁡(At).

This approximation is valid under the assumption (2.4), which is true when the number of diffusive molecules and the value of diffusion coefficient are large. Overall, we approximate the numbers of diffusive jumps by obtaining (i) the macroscopic features of the diffusive jumps, *Φ*:=(*ϕ*_1*R*_,*ϕ*_2*L*_,*ϕ*_2*R*_,…,*ϕ*_*KL*_)′; (ii) the distributions of the fluctuations, *Ξ*:=(*ξ*_1*L*_,*ξ*_2*L*_,*ξ*_2*R*_,…,*ξ*_*KL*_)′ and (iii) the probability, *P*_R_(*t*), that no reaction will occur until time *t*. These three components can be solved as follows:

1. *Φ* satisfies the following equations:
  2.6 $$\begin{array}{rl} & \frac{d\Phi }{dt}=A\Omega +\left(\frac{D_{1}}{h^{2}}\right)C\\ \;\text{and}\;\; & \Omega =0\;\text{at }t=t_{0}, \end{array}\}$$
  where *C*=(*x*_0_11__,*x*_0_21__,…,*x*_0_*k*1__,…,*x*_0_*K*1__)′/*Λ*. Based on the study of Othmer & Scriven [30], the eigenvalues and eigenvectors of *A* can be obtained analytically and used to solve the system directly.
2. *Ξ* is a Gaussian random vector with a mean of zero and a covariance of
  2.7 $$\rho =\int_{0}^{t}Y(t)Y^{-1}(\tau )B(\tau ){\left(Y^{-1}(\tau )\right)}^{\prime }Y{\left(t\right)}^{\prime }\;d\tau ,$$
  where Y(t)=exp⁡(At) and *B*(*t*)=*diag*(∂*Φ*/∂*t*) is a diagonal matrix with the diagonal elements equal to ∂*Φ*/∂*t*.
3. *P*_R_(*t*) takes the form
  $$P_{R}(t)=\exp\left(-\int_{t_{0}}^{t}\sum_{l=1}^{K}\sum_{j=1}^{M}a_{lj}\left(x_{0}+\Lambda \sum_{k=1}^{K-1}\phi_{kR}(\tau )v_{kR}+\Lambda \sum_{k=2}^{K}\phi_{kL}(\tau )\nu_{kL}\right)d\tau \right),$$
  where *a*_*lj*_ is the propensity function of reaction *R*_*lj*_ for *l*=1,2,…,*K* and *j*=1,2,…,*M*.

### 2.3. Adaptivity of the time step and the spatial partitioning of the compartments

To determine the next time step after time *t*_0_, we generate a random number *r* that is uniformly distributed in [0,1] and find *t* such that *P*_R_(*t*)=*r*. To update the numbers of diffusive jumps at time *t*, we calculate *Φ* and generate *Ξ*, a random vector that has a multivariate normal distribution with a mean of zero and a covariance of *ρ*. However, because *t* is chosen as a random number, the interval between *t* and *t*_0_ might be short; this would allow only a small number of diffusive jumps to occur among compartments with a low copy number of molecules, leading to poor accuracy of the Gaussian approximation. In such compartments where the Gaussian approximation fails due to a low copy number of molecules, we simulate the diffusive jumps using the SSA method. To determine which compartments fit this criterion, we compare the Poisson distribution with mean *μ* to the Gaussian distribution with mean *μ* and variance *μ*. It is known that as *μ* increases, the difference between the two distributions decreases. For example, when *μ* is larger than 10, we have

2.8 $$\max_{n\geq 0}\left|\frac{1}{\sqrt{2\pi \mu }}\exp\left[-\frac{{\left(n-\mu \right)}^{2}}{2\mu }\right]-\frac{\exp(-\mu )\mu^{n}}{n!}\right|\leq 0.01.$$

Therefore, we apply the SSA method for diffusion in those compartments where the mean number of diffusive jumps is small (e.g. fewer than 10). For the case in which the amount of species *S*_1_ decreases monotonically towards the *K*th compartment in the deterministic model, we use the SSA method in all compartments with an index larger than or equal to *k*_*g*_(*t*), which is the smallest integer such that

2.9 $$\Lambda \phi_{k_{g}(t)L}(t)\leq T_{A}\;\text{or}\;\Lambda \phi_{k_{g}(t)R}(t)\leq T_{A},$$

where *Λϕ* approximates the mean value of diffusive jump and *T*_*A*_ is the cut-off threshold for determining the region for the Gaussian approximation. We set *T*_*A*_=10 to ensure that the absolute difference between the Poisson and Gaussian distributions is less than 0.01, as seen in (2.8). When *T*_*A*_ increases, the SSA is applied in more compartments and it may increase the CPU time cost in our hybrid method. For two or more types of diffusive molecules, we can still apply (2.9) as long as the diffusion process of each type of molecules is independent of each other. As a result, different types of diffusive molecules have different *k*_*g*_ values.

### 2.4. Algorithm overview

Given *x*(*t*_0_)=*x*_0_, we perform the following steps:

1. Generate two independent random numbers *r*_1_ and *r*_2_ that are uniformly distributed in [0,1].
2. Find *t* such that *P*_R_(*t*)=*r*_1_
  and let
  $${\bar{\alpha }}_{lj}=\int_{t_{0}}^{t}a_{lj}\left(x_{0}+\Lambda \sum_{k=1}^{K-1}\phi_{kR}(\tau )v_{kR}+\Lambda \sum_{k=2}^{K}\phi_{kL}(\tau )\nu_{kL}\right)d\tau .$$
3. Evaluate *k*_*g*_ using equation (2.9) to determine the compartments for which the SSA method will be used. If *k*_*g*_=1, simulate diffusion in all compartments using the SSA method; if *k*_*g*_>1, perform the following steps:
  (a) simulate diffusion between the *k*_*g*_th compartment and the *K*th compartment using the SSA method, and treat the *k*_*g*_th compartment as a reflective boundary;
  (b) calculate *Φ* and *ρ* in equation (2.6) and equation (2.7) at time *t*; remove rows 2*k*_*g*_−2,2*k*_*g*_−1,…,2*K*−2 and columns 2*k*_*g*_−2,2*k*_*g*_−1,…,2*K*−2 in the matrix *ρ*, and let *ρ*_*s*_ be the remaining submatrix;
  (c) generate a (2*k*_*g*_−3)-variate Gaussian random vector *Ξ* with a mean of zero and a covariance of *ρ*_*s*_;
  (d) evaluate *n*_*kL*_ and *n*_*kR*_ in equations (2.2) and (2.3) at time *t*, and update *x*_*k*1_ using *n*_*kL*_ and *n*_*kR*_ for 1≤*k*≤*k*_*g*_−2;
  (e) add *n*_(*k*_*g*_−2)*R*_−*n*_(*k*_*g*_−1)*L*_−*n*_(*k*_*g*_−1)*R*_+*Λϕ*_*k*_*g*_*L*_ to *x*_(*k*_*g*_−1)1_, and add *n*_(*k*_*g*_−1)*R*_−*Λϕ*_*k*_*g*_*L*_ to *x*_*k*_*g*_1_; and
  (f) round the *x*_*k*1_ values to their nearest non-negative integers.
4. Find the smallest values of *m* and *q* such that
  $$\sum_{l=1}^{q-1}\sum_{j=1}^{M}{\bar{\alpha }}_{lj}+\sum_{j=1}^{m}{\bar{\alpha }}_{qj}\geq r_{2}\sum_{l=1}^{K}\sum_{j=1}^{M}{\bar{\alpha }}_{lj}.$$
  Then, let the *m*th reaction occur in the *q*th compartment, and update *x* in accordance with reaction *R*_*qm*_.
5. Advance the time to *t*. Then, go back to step 1 until the simulation time reaches the stop criterion.

In this algorithm, the calculations of ${\bar{\alpha }}_{lj}$ in step 2 and *ρ* in step 3(b) are the most computationally expensive steps among all steps. ${\bar{\alpha }}_{lj}$ and *ρ* can be estimated using simple numerical integration quadratures such as the trapezoidal rule, which we use in the test cases presented below. In step 3(b), *Φ* can be solved directly and efficiently using the eigenvalues and eigenvectors of *A*. Negative values and non-integers may appear in the Gaussian approximation so we round the values to their nearest non-negative integers in step 3(f). This step may lead to loss of mass. However, the error introduced by this rounding can be reduced by increasing the number of molecules.

## 3. Simulation results

In this section, we will compare the performance of the hybrid method and the SSA using different morphogen models. First a one-dimensional simple morphogen system with different values of diffusion coefficients is considered. Next, we extend our study to a morphogen–receptor system, a morphogen system containing two types of diffusive molecules, a two-dimensional morphogen system and a Turing system to show that the results are consistent with the one-dimensional simple morphogen system we consider in the first part. All the numerical tests are implemented in Matlab.

### 3.1. Example I: morphogen system with linear degradation

**Figure 1.**
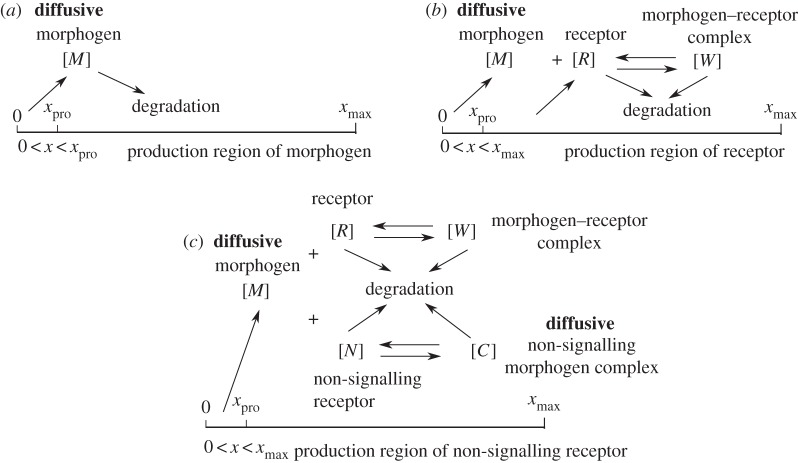
Morphogen models used for numerical tests in examples I–IV. (*a*) Examples I and IV: morphogen system with linear degradation. (*b*) Example II: morphogen–receptor system. (*c*) Example III: morphogen system with two types of diffusive molecules.

First, we consider a one-dimensional morphogen system in which morphogens diffuse out from a local production region and undergo degradation throughout the entire domain (figure 1*a*). This model was used to study the stochastic effect on patterning of the *Drosophila* wing disc [31,32]. The deterministic dynamics of morphogen concentration [*M*] can be described by the following PDE system with no-flux boundary conditions:

$$\frac{\partial [M]}{\partial t}=D_{M}\frac{\partial^{2}[M]}{\partial x^{2}}-d_{M}[M]+V(x),$$

where *D*_M_ is the diffusion coefficient, *d*_M_ is the degradation rate coefficient and *V* (*x*) is the production rate of morphogen. We define the production rate as

$$V(x)=v_{M}\;\text{if }0\leq x\leq x_{\mathrm{pro}};\;V(x)=0\;\text{if }x_{\mathrm{pro}}<x\leq x_{\max}.$$

The one-dimensional domain $\left[0,x_{\max}\right]$ is divided into 100 compartments with 2 μm width which is based on the cell size of the Drosophila wing disc, so that the domain size we consider is $x_{\max}=200\;\text{μ}m$. The initial condition for simulations is [*M*](0,*x*)=*V* (*x*)/*d*_M_. The parameters are listed in table 1.

**Table 1.** List of parameter values used in examples I–IV. The values are based on [32].

parameters	definitions	values
*D*_M_	diffusion coefficient of free morphogens	5 μm^2^ s^−1^−40 μm^2^ s^−1^
*D*_C_	diffusion coefficient of morphogen–receptor complexes	5 μm^2^ s^−1^−10 μm^2^ s^−1^
*v*_M_	production rate of morphogens	10^−4^ μM s^−1^
*v*_R_	production rate of receptors	10^−4^ μM s^−1^
*v*_N_	production rate of non-signalling receptors	2×10^−4^ μM s^−1^
*d*_M_	degradation rate coefficient of morphogens	10^−2^ s^−1^
*d*_R_	degradation rate coefficient of receptors	10^−4^ s^−1^
*d*_N_	degradation rate coefficient of non-signalling receptors	10^−4^ s^−1^
*d*_W_	degradation rate coefficient of receptors	10^−4^ s^−1^
*d*_C_	degradation rate coefficient of non-signalling receptors	10^−4^ s^−1^
*α*_1_	binding rate coefficient for morphogen–receptor complexes	5×10^−2^ μM s^−1^
*β*_1_	dissociation rate coefficient for morphogen–receptor complexes	5×10^−4^ μM s^−1^
*α*_2_	binding rate coefficient for morphogen–non-receptor complexes	5×10^−2^ μM s^−1^
*β*_2_	dissociation rate coefficient for morphogen–non-receptor complexes	5×10^−4^ μM s^−1^
*Λ*_M_	number of morphogens per unit concentration in a compartment	1.8×10^4^ μM^−1^
*Λ*_R_ (*Λ*_N_)	number of receptors (non-receptors) per unit concentration in a compartment	2×10^2^ μM^−1^
*x*_pro_	size of morphogen production region	14 μm

**Figure 2.**
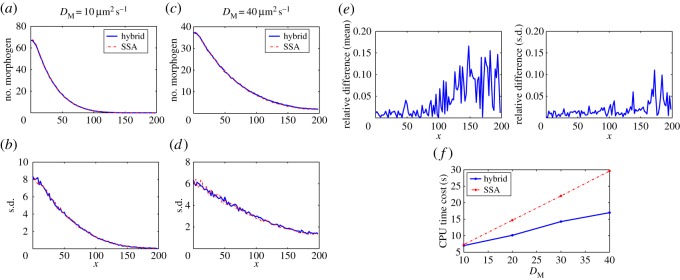
Simulation results for example I with different values of *D*_M_. In the results, 500 simulations were used to calculate the statistical quantities for each case. The parameters are listed in table 1. (*a*,*b*) Model with diffusion coefficient 10 μm^2^ s^−1^: (*a*) means of the number of morphogens and (*b*) standard deviations of the number of morphogens. (*c*–*d*) Model with diffusion coefficient 40 μm^2^ s^−1^: (*c*) means of the number of morphogens and (*d*) standard deviations of the number of morphogens. (*e*) Relative differences of means and standard deviations for the simulations with diffusion coefficient 40 μm^2^ s^−1^. (*f*) CPU time costs for two stochastic methods with different values of diffusion coefficients.

Figure 2*a*–*d* displays the means and standard deviations of the number of morphogens in each compartment at simulation time 100 s, based on both the SSA and the hybrid method with different diffusion coefficients *D*_M_=10 μm^2^ s^−1^ and *D*_M_=40 μm^2^ s^−1^. In the results, 500 simulations were used to calculate the statistical quantities for each case.

As observed in figure 2*a*–*d*, there is good agreement between the results of the two methods throughout the simulations. For systematic comparison, we calculate the relative differences between the simulations by the SSA and the hybrid method, (*M*_SSA_−*M*_Hybrid_)/*M*_SSA_. Figure 2*e* shows that the relative differences of means and standard deviations are less than 0.15 when *D*_M_=40 μm^2^ s^−1^. Then we calculate the averages of CPU time costs in the 500 simulations of each case. Figure 2*f* shows that the CPU time cost of the SSA is linearly increasing with the value of *D*_M_, and is much higher than the cost of the hybrid method when *D*_M_=40 μm^2^ s^−1^. Although the CPU time cost of the hybrid method is also increasing with the value of *D*_M_, the increasing rate is much lower than the SSA. When the diffusion coefficient *D*_M_ increases, the morphogen system becomes a diffusion-dominant system that highlights the advantage of the diffusion approximation which allows a larger time step for each time iteration in our hybrid method.

**Figure 3.**
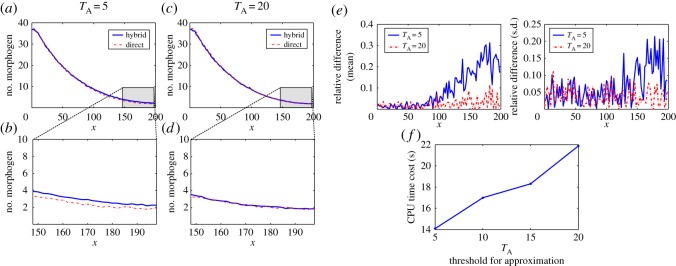
Simulation results for example I with different values of *T*_*A*_ in (2.9). In the results, 500 simulations were used to calculate the statistical quantities for each case. The parameters are listed in table 1 and *D*_M_= 40 μm^2^ s^−1^. (*a*–*b*) Model with *T*_*A*_=5: (*a*) means of the number of morphogens and (*b*) means of the number of morphogens in the region of lower copy number of morphogens. (*c*–*d*) Model with *T*_*A*_=20: (*c*) means of the number of morphogens and (*d*) means of the number of morphogens in the region of lower copy number of morphogens. (*e*) Relative differences of means and standard deviations for the simulations with *T*_*A*_=5 and *T*_*A*_=20. (*f*) CPU time costs for the hybrid methods with different values of *T*_*A*_.

Other than changing the value of the diffusion coefficient *D*_M_, we test how the value of *T*_*A*_ in (2.9) affects the CPU time cost and the performance of the hybrid method. For the simulations in figure 3, we apply the setting used in figure 2, but with different values of *T*_*A*_. The simulations in figure 3*a*–*d* show that the accuracy of the hybrid method improves when *T*_*A*_ increases. Figure 3*e* displays that the relative differences of means and standard deviations between the simulations by the hybrid method and the SSA decrease significantly in the region of low copy number of molecules when *T*_*A*_ increases from 5 to 20. By contrast, figure 3*f* shows a trade-off in which the CPU time cost is increasing with the value of *T*_*A*_.

### 3.2. Example II: morphogen–receptor system

Here we consider a one-dimensional morphogen–receptor system in which receptors are produced in the entire region and bind with morphogen to induce morphogen degradation (figure 1*b*). This model involves the stochastic effect in a binding process between two kinds of molecules [32,33]. The deterministic dynamics of three kinds of molecule concentrations (morphogen [*M*], receptor [*R*] and morphogen–receptor complex [*W*]) can be described by the following PDE system with no-flux boundary conditions:

$$\begin{array}{rl} \frac{\partial [M]}{\partial t} & =D_{M}\frac{\partial^{2}[M]}{\partial x^{2}}-\alpha_{1}[M][R]+\beta_{1}[W]+V(x),\\ \frac{\partial [R]}{\partial t} & =-\alpha_{1}[M][R]+\beta_{1}[W]-d_{R}[R]+v_{R},\\ \;\mathrm{and}\;\frac{\partial [W]}{\partial t} & =\alpha_{1}[M][R]-\beta_{1}[W]-d_{W}[W]. \end{array}$$

The morphogen production function *V* (*x*), the domain and its discretization are defined as in example I. The initial conditions for simulations are [*M*](0,*x*)=*V* (*x*)/*d*_M_, [*R*](0,*x*)=*V*_R_/*d*_R_ and [*W*](0,*x*)=0. The parameters are listed in table 1.

**Figure 4.**
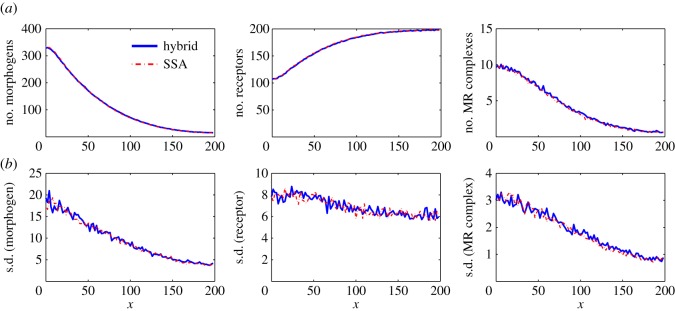
Simulation results for example II. (*a*) Means of the numbers of morphogens, receptors and morphogen–receptor complexes. (*b*) The corresponding standard deviations. In the results, 200 simulations were used to calculate the statistical quantities for each case. In this simulation, we take *D*_M_= 5 μm^2^ s^−1^ and the remaining parameters are listed in table 1.

Figure 4 displays the means and standard deviations of the numbers of morphogens, receptors and morphogen–receptor complexes in each compartment at simulation time 1000 s, based on both the SSA and the hybrid method with *D*_M_=5 μm^2^ s^−1^. In this case, 200 simulations were used to calculate the statistical quantities. The means calculated using both stochastic methods agree well with each other, and the standard deviations are consistent between the two stochastic methods. The simulation results with *D*_M_=10 μm^2^ s^−1^ and *D*_M_=20 μm^2^ s^−1^ also have good agreement between the SSA and the hybrid method. For studying time cost, we calculate the averages of CPU time costs in the 200 simulations of each case. Table 2 shows that the CPU time cost of the SSA is much higher than the cost of the hybrid method when *D*_M_=5 μm^2^ s^−1^, 10 μm^2^ s^−1^ or 20 μm^2^ s^−1^. In particular, the CPU time cost of the hybrid method is around 20% of that of the SSA when *D*_M_=20 μm^2^ s^−1^.

**Table 2.** CPU time cost of SSA and hybrid methods for examples II–III.

	example II			example III		
*D*_M_(μm^2^ s^−1^)	5	10	20	5	10	20
*D*_C_(μm^2^ s^−1^)	—	—	—	5	10	20
CPU time (SSA)	251.6 s	495.5 s	949.8 s	757.0 s	1481.2 s	2868.4 s
CPU time (hybrid)	126.3 s	177.7 s	187.6 s	457.8 s	703.6 s	926.7 s

### 3.3. Example III: morphogen system with two types of diffusive molecules

In this example, we want to study the performance of the hybrid method for the system with two types of diffusive molecules. Many studies on morphogen system were based on reaction–diffusion models with only one diffusion term in either free or non-signalling bound-morphogens [31–33]. However, this type of one-diffusion model is not biologically complete because it is possible to have more than one type of diffusion transport. Morphogen models with two types of diffusive molecules have been proposed and studied in [34–36]. Here we consider a morphogen model with both diffusion of free morphogens and movement of non-signalling morphogen complexes through a ‘bucket brigade’ process [35] (figure 1*c*). The deterministic dynamics of five kinds of molecule concentrations (morphogen [*M*], receptor [*R*], non-signalling receptor [*N*], morphogen–receptor complex [*W*] and diffusive non-signalling morphogen complex [*C*]) can be described by the following PDE system with no-flux boundary conditions:

$$\begin{array}{rl} \frac{\partial [M]}{\partial t} & =D_{M}\frac{\partial^{2}[M]}{\partial x^{2}}-\alpha_{1}[M][R]+\beta_{1}[W]-\alpha_{2}[M][N]+\beta_{2}[C]+V(x),\\ \frac{\partial [R]}{\partial t} & =-\alpha_{1}[M][R]+\beta_{1}[W]-d_{R}[R]+v_{R},\\ \frac{\partial [W]}{\partial t} & =\alpha_{1}[M][R]-\beta_{1}[W]-d_{W}[W],\\ \frac{\partial [N]}{\partial t} & =-\alpha_{2}[M][N]+\beta_{2}[C]-d_{N}[N]+v_{N},\\ \;\text{and}\;\frac{\partial [C]}{\partial t} & =D_{C}\frac{\partial^{2}[C]}{\partial x^{2}}+\alpha_{2}[M][N]-\beta_{2}[C]-d_{C}[C]. \end{array}$$

Similar to the previous examples, the morphogen production function *V* (*x*), the domain and its discretization are defined as in example I. The initial conditions for simulations are [*M*](0,*x*)=*V* (*x*)/*d*_M_, [*R*](0,*x*)=*V*_R_/*d*_R_, [*W*](0,*x*)=0, [*N*](0,*x*)=*V*_N_/*d*_N_ and [*C*](0,*x*)=0. The parameters are listed in table 1.

**Figure 5.**
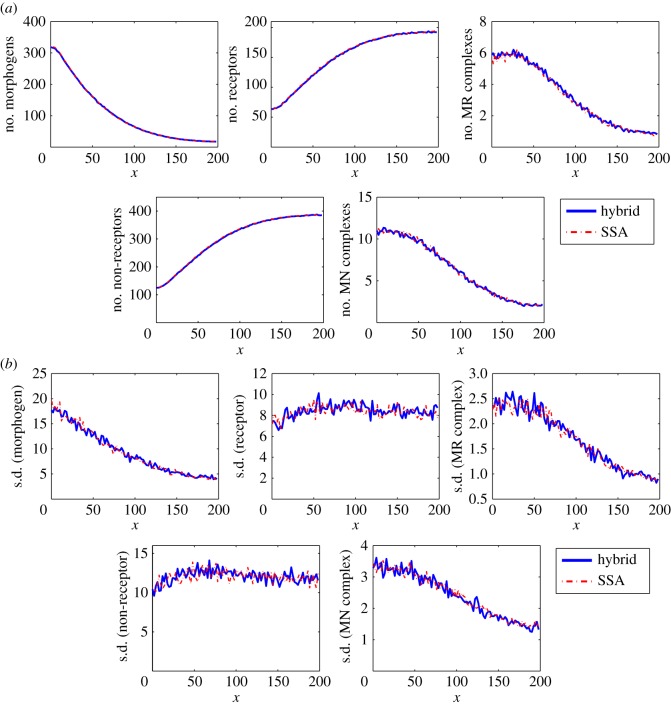
Simulation results for example III. (*a*) Means of the numbers of morphogens, receptors, morphogen–receptor complexes, non-signalling receptors and diffusive non-signalling morphogen complexes. (*b*) The corresponding standard deviations. In the results, 200 simulations were used to calculate the statistical quantities. In this simulation, we take $D_{M}=D_{C}=5\;\text{μ}m^{2}\;s^{-1}$ and the remaining parameters are listed in table 1.

Figure 5 reveals that the statistical quantities obtained using the hybrid method agree well with those obtained using the SSA with diffusion coefficients *D*_M_=*D*_C_=5 μm^2^ s^−1^ at simulation time 2000 s. Table 2 displays the CPU cost of the hybrid method is only 60%, 48% and 32% of that of the SSA when the diffusion coefficients are $D_{M}=D_{C}=5\;\text{μ}m^{2}\;s^{-1}$, $D_{M}=D_{C}=10\;\text{μ}m^{2}\;s^{-1}$ and $D_{M}=D_{C}=20\;\text{μ}m^{2}\;s^{-1}$, respectively. Our hybrid method works very well for such systems with more than one type of diffusive molecules.

### 3.4. Example IV: two-dimensional morphogen system

Here, we study the performance of hybrid method for a two-dimensional morphogen system which is based on example I (figure 1*a*). The two-dimensional domain $\left[0,x_{\max}]\times [-y_{\max},y_{\max}\right]$ is divided into 250 compartments with uniform dimension 2×2 μm, which is based on the cell size of Drosophila wing disc. The domain size we consider is $x_{\max}=100\;\text{μ}m$ and $y_{\max}=5\;\text{μ}m$. We define the morphogen production function as

3.1 $$V(x,y)=v_{M}\;\text{if }0\leq x\leq x_{\mathrm{pro}};\;V(x)=0\;\text{if }x_{\mathrm{pro}}<x\leq x_{\max}.$$

The initial condition for simulations is [*M*](0,*x*,*y*)=*V* (*x*,*y*)/*d*_M_. The parameters for this example are equal to the set we used in example I. Five hundred simulations were used to calculate the statistical quantities. In this case, the number of morphogens decreases monotonically along the *x*-axis in the deterministic model, so the condition (2.9) is applied on *x*-direction.

**Figure 6.**
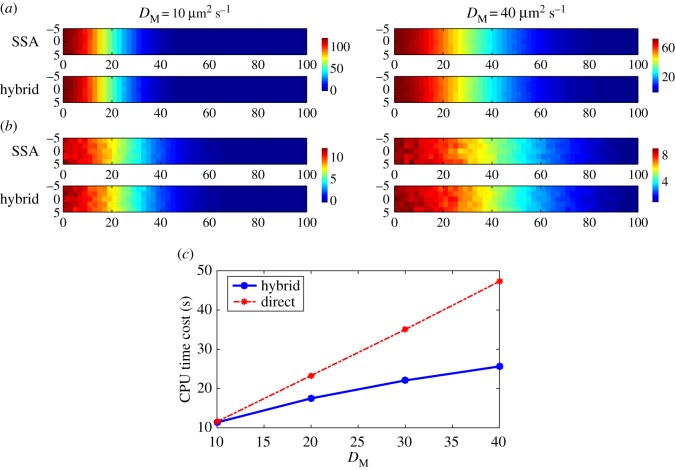
Simulation results for example IV. (*a*) Means of the numbers of morphogens for the system with *D*_M_=5 μm^2^ s^−1^ and *D*_M_= 40 μm^2^ s^−1^. (*b*) The corresponding standard deviations. (*c*) CPU time costs for different methods and different values of diffusion coefficients. In the results, 200 simulations were used to calculate the statistical quantities for each case. Other than the diffusion coefficient, the remaining parameters are listed in table 1.

Figure 6*a*,*b* displays the means and standard deviations of the number of molecules in each 2 μm×2 μm compartment at simulation time 10 s, based on both the SSA and the hybrid method with different diffusion coefficients *D*_M_=10 μm^2^ s^−1^ and *D*_M_=40 μm^2^ s^−1^. The figures support that the two stochastic methods have good agreement in two-dimensional simulations. This result is consistent with the one-dimensional simulations shown in figure 2. The CPU time cost of the SSA is linearly increasing with the value of *D*_M_, and the cost of the hybrid method is around 50% of the SSA when *D*_M_=40 μm^2^ s^−1^ (figure 6*c*).

### 3.5. Example V: non-monotone pattern

**Figure 7.**
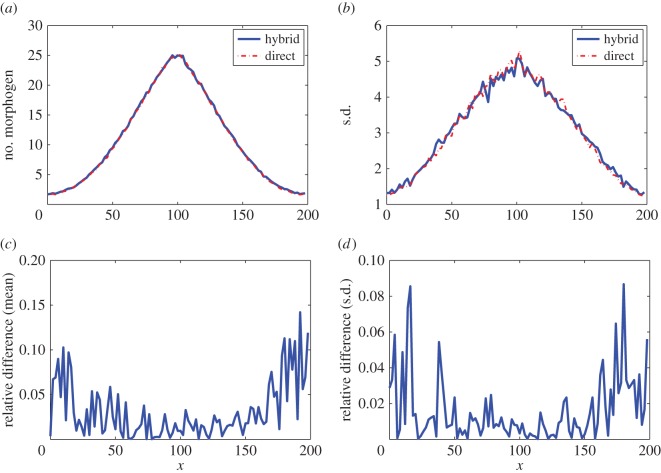
Simulation results for example V in which the morphogen production region is located at the centre of the domain. In the results, 500 simulations were used to calculate the statistical quantities for each case. The parameters are listed in table 1 and *D*_M_=40 μm^2^ s^−1^. (*a*) Means of the number of morphogens. (*b*) Standard deviations of the number of morphogens. (*c*) Relative differences of means for the simulations by the SSA and the hybrid method. (*d*) Relative differences of standard deviations for the simulations by the SSA and the hybrid method.

In general, the adaptive method can be applied for some types of non-monotone pattern. Similar to example I, when the morphogen production region is considered at the centre of the domain as

$$V(x)=v_{M}\;\text{if }94\leq x\leq 106;\;V(x)=0\text{ otherwise},$$

in the one-dimensional domain [0,200], the same setting used in example I can generate a single-peak solution as in figure 7*a*. We reformulate the condition (2.9) by considering the largest region [*k*_*g*1_,*k*_*g*2_] near the peak such that for all *j*∈[*k*_*g*1_,*k*_*g*2_], we have

3.2 $$\Lambda \phi_{jL}(t)>T_{A}\;\text{or}\;\Lambda \phi_{jR}(t)>T_{A}.$$

This condition ensures the accuracy of the Gaussian approximation in the region at the centre. Figure 7*a*,*b* displays the means and standard deviations of the number of molecules in each compartment at simulation time 20 s with *D*=40 μm^2^ s^−1^. In the results, 500 simulations were used to calculate the statistical quantities for each case. Figure 7*c*,*d* shows that the relative differences of means and standard deviations between the simulations by the hybrid method and the SSA are less than 0.15. For efficiency, the CPU time cost of the hybrid method is around 57% of that of the SSA. The adaptive method can be extended to patterns consisting of multiple peaks if the peaks can be determined by the deterministic system.

### 3.6. Example VI: Turing system

In the last example, we consider an activator-substrate Turing system consisting of a short-range diffusion for the activator and a long-range diffusion for the substrate. The normalized one-dimensional activator-substrate Turing system [37] can be described by the following two-equation system with the no-flux boundary conditions:

$$\begin{array}{rl} \frac{\partial [A]}{\partial t} & =D_{A}\frac{\partial^{2}[A]}{\partial x^{2}}+\alpha [S]{\left[A\right]}^{2}-\beta [A]+\rho_{A},\\ \frac{\partial [S]}{\partial t} & =D_{S}\frac{\partial^{2}[S]}{\partial x^{2}}-\alpha [S]{\left[A\right]}^{2}+\rho_{S}, \end{array}$$

in 0≤*x*≤10. The constants *D*_A_ and *D*_S_ measure the diffusion coefficients of activators and substrates, respectively. In order to generate a spatial inhomogeneous steady-state solution, the diffusion coefficient *D*_A_ needs to be much less than *D*_S_. Here we use *D*_S_=50 and *D*_A_=0.1. For other parameters, we take *α*=*β*=*ρ*_S_=1 and *ρ*_A_=0.01. The one-dimensional domain [0,10] is divided into 50 compartments. The number of molecules per unit concentration in a compartment is *Λ*_A_=*Λ*_S_=500. The initial condition for simulations is the homogeneous steady-state solution [*A*](0,*x*)=1.01 and [*S*](0,*x*)=(1.01)^−2^.

The Gaussian approximation is used only for the fast diffusion in substrates. Since the chemical gradients may not be monotonic in space, the time-adaptive criterion (2.9) is not used in this example, and instead, the Gaussian approximation for the diffusion process of substrates is applied everywhere in space. Since the number of substrates is large enough in each compartment, the approximation has a good accuracy in the entire space.

**Figure 8.**
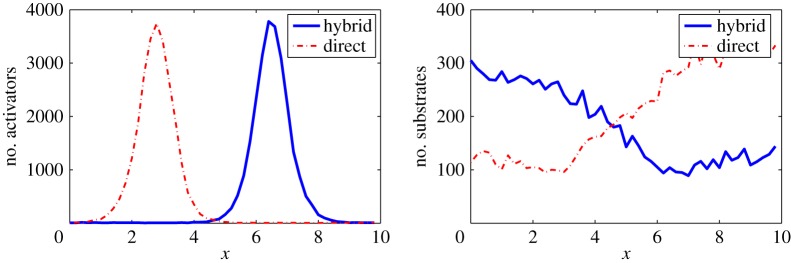
Example simulations obtained by the SSA (dashed line)and the hybrid method (solid line) for the one-dimensional activator-substrate Turing system in example VI. In the hybrid method, the Gaussian approximation is applied for simulating the diffusion processes of substrates which have rapid diffusion processes.

The study using different methods (figure 8) indicates that two stochastic methods show a similar pattern for *A* at *t*=10. The averages of CPU time costs in the 500 simulations for different methods indicate that the SSA costs 5.61×10^3^ s for each simulation and the hybrid method costs 2.16×10^3^ s, suggesting a 50% speed-up of the hybrid method.

## 4. Discussion

We have introduced a new algorithm to accelerate the stochastic simulation of reaction–diffusion systems. In this hybrid approach, the numbers of diffusive jumps in regions with a high level of molecules are calculated using Gaussian vectors, whereas the diffusive jumps in regions with a low level of molecules, along with the reaction events, are simulated using the SSA. Because of the diffusion approximation, the size of the time step, which predominantly depends on rate of reactions, can be significantly larger than that allowed by the existing approaches. Thus, the hybrid method is particularly effective for diffusion-dominant systems. Moreover, the diffusion approximations for different diffusing species are performed independently, which makes the method particularly advantageous for application to systems with multiple diffusing species.

To determine the compartments for which the diffusion approximation is applied, we use a macroscopic quantity that can be easily calculated. In addition, the approximation region can be adaptively specified in both space and time with a good control of approximation errors. Thus, the hybrid method is effective in dealing with spatial distributions of molecules that may vary significantly over time or may not be monotonic in space.

The Gaussian random vector is directly related to the matrix whose non-zero location is determined by the communication among compartments. The partitioning of a domain with an irregular geometry may be achieved using existing software (COMSOL or URDME [38]) to enable application to complex domains. To extend our method for the system incorporating rapid and slow reactions, we can accelerate the simulation of rapid reactions by coupling our method with the hybrid SSA/*τ*-Leaping strategy [6] to create a hybrid diffusion approximation + SSA/*τ*-Leaping strategy. The overall approach introduced in this work will be an efficient and accurate algorithm for simulating biological and physical systems in which number of molecular copies has a large range within the spatial domain and diffusion is dominant comparing with other types of reactions.

## Appendix A

**A.1. Spatial stochastic simulation algorithm**

The stochastic process underlying the CME can be simulated by the SSA. In SSA, two random numbers *r*_1_ and *r*_2_, that are uniformly distributed in [0, 1], are generated for determining the time and the index of the next reaction, respectively. The time for the next reaction is *t*+*τ*, with

$$\tau =-\frac{\ln r_{1}}{a_{0}(x)},$$

where *a*_0_(*x*) is the sum of all propensity functions:

$$a_{0}(x)=\sum_{k=1}^{K}\sum_{j=1}^{M}a_{kj}(x)+\sum_{k=2}^{K}a_{kL}(x)+\sum_{k=1}^{K-1}a_{kR}(x).$$

The index of the next reaction is obtained by finding the smallest *m* and *q* such that

A 1 $$\begin{array}{rl} & \sum_{k=1}^{q-1}\sum_{j=1}^{M}a_{kj}+\sum_{j=1}^{m}a_{qj}\geq r_{2}a_{0} \end{array}$$

A 2 $$\begin{array}{rl} \text{or}\; & \sum_{k=1}^{K}\sum_{j=1}^{M}a_{kj}+\sum_{k=2}^{q}a_{kL}\geq r_{2}a_{0} \end{array}$$

A 3 $$\begin{array}{rl} \text{or}\; & \sum_{k=1}^{K}\sum_{j=1}^{M}a_{kj}+\sum_{k=2}^{K}a_{kL}+\sum_{k=1}^{q}a_{kR}\geq r_{2}a_{0}. \end{array}$$

If the condition (A 1) is satisfied, then the reaction *R*_*qm*_ happens and the state of the system is updated as *X*(*t*+*τ*)=*X*(*t*)+*ν*_*qm*_; or if the condition (A 2) is satisfied, then the diffusion jump *J*_*qL*_ happens and the state of the system is updated as *X*(*t*+*τ*)=*X*(*t*)+*ν*_*qL*_; or if the condition (A 3) is satisfied, then the diffusion jump *J*_*qR*_ happens and the state of the system is updated as *X*(*t*+*τ*)=*X*(*t*)+*ν*_*qR*_.

The simulation repeats the above process until it reaches the stop criterion.

## References

[1] Balázsi G, van Oudenaarden A, Collins JJ 2011 Cellular decision making and biological noise: from microbes to mammals. *Cell* 144, 910–925. (doi:10.1016/j.cell.2011.01.030) 21414483 10.1016/j.cell.2011.01.030 PMC3068611

[2] Eldar A, Elowitz MB 2010 Functional roles for noise in genetic circuits. *Nature* 467, 167–173. (doi:10.1038/nature09326) 20829787 10.1038/nature09326 PMC4100692

[3] Fange D, Elf J 2006 Noise-induced min phenotypes in *E. coli*. *PLoS Comput. Biol.* 2, e80 (doi:10.1371/journal.pcbi.0020080) 16846247 10.1371/journal.pcbi.0020080 PMC1484588

[4] Gillespie DT 1976 A general method for numerically simulating the stochastic time evolution of coupled chemical reactions. *J. Comput. Phys.* 22, 403–434. (doi:10.1016/0021-9991(76)90041-3)

[5] Gibson MA, Bruck J 2000 Efficient exact stochastic simulation of chemical systems with many species and many channels. *J. Phys. Chem. A* 104, 1876–1889. (doi:10.1021/jp993732q)

[6] Cao Y, Gillespie DT, Petzold LR 2005 Avoiding negative populations in explicit Poisson tau-leaping. *J. Chem. Phys.* 123, 054104 (doi:10.1063/1.1992473) 16108628 10.1063/1.1992473

[7] Cao Y, Gillespie DT, Petzold LR 2007 Adaptive explicit-implicit tau-leaping method with automatic tau selection. *J. Chem. Phys.* 126, 224101 (doi:10.1063/1.2745299) 17581038 10.1063/1.2745299

[8] Gillespie DT 2001 Approximate accelerated stochastic simulation of chemically reacting systems. *J. Chem. Phys.* 115, 1716–1733. (doi:10.1063/1.1378322)

[9] Cao Y, Gillespie DT, Petzold LR 2005 The slow-scale stochastic simulation algorithm. *J. Chem. Phys.* 122, 014116 (doi:10.1063/1.1824902) 10.1063/1.1824902 15638651

[10] Haseltine EL, Rawlings JB 2002 Approximate simulation of coupled fast and slow reactions for stochastic chemical kinetics. *J. Chem. Phys.* 117, 6959–6969. (doi:10.1063/1.1505860)

[11] Liu Z, Pu Y, Li F, Shaffer CA, Hoops S, Tyson JJ, Cao Y 2012 Hybrid modeling and simulation of stochastic effects on progression through the eukaryotic cell cycle. *J. Chem. Phys.* 136, 034105 (doi:10.1063/1.3677190) 22280742 10.1063/1.3677190 PMC3272065

[12] Lester C, Yates CA, Giles MB, Baker RE 2015 An adaptive multi-level simulation algorithm for stochastic biological systems. *J. Chem. Phys.* 142, 024113 (doi:10.1063/1.4904980) 25591344 10.1063/1.4904980

[13] Gardiner C, McNeil K, Walls D, Matheson I 1976 Correlations in stochastic theories of chemical reactions. *J. Stat. Phys.* 14, 307–331. (doi:10.1007/BF01030197)

[14] Isaacson SA, Peskin CS 2006 Incorporating diffusion in complex geometries into stochastic chemical kinetics simulations. *SIAM J. Sci. Comput.* 28, 47–74. (doi:10.1137/040605060)

[15] Kang HW, Zheng L, Othmer HG 2012 A new method for choosing the computational cell in stochastic reaction–diffusion systems. *J. Math. Biol.* 65, 1017–1099. (doi:10.1007/s00285-011-0469-6) 22071651 10.1007/s00285-011-0469-6 PMC3765051

[16] Erban R, Chapman SJ 2009 Stochastic modelling of reaction–diffusion processes: algorithms for bimolecular reactions. *Phys. Biol.* 6, 046001 (doi:10.1088/1478-3975/6/4/046001) 19700812 10.1088/1478-3975/6/4/046001

[17] Isaacson SA 2009 The reaction-diffusion master equation as an asymptotic approximation of diffusion to a small target. *SIAM J. Appl. Math.* 70, 77–111. (doi:10.1137/070705039)

[18] Elf J, Ehrenberg M 2004 Spontaneous separation of bi-stable biochemical systems into spatial domains of opposite phases. *Syst. Biol.* 1, 230–236. (doi:10.1049/sb:20045021) 10.1049/sb:20045021 17051695

[19] Hanusse P, Blanché A 1981 A Monte Carlo method for large reaction–diffusion systems. *J. Chem. Phys.* 74, 6148–6153. (doi:10.1063/1.441005)

[20] Hu J, Kang HW, Othmer HG 2014 Stochastic analysis of reaction-diffusion processes. *Bull. Math. Biol.* 76, 854–894. (doi:10.1007/s11538-013-9849-y) 23719732 10.1007/s11538-013-9849-y PMC3825834

[21] Lampoudi S, Gillespie DT, Petzold LR 2009 The multinomial simulation algorithm for discrete stochastic simulation of reaction-diffusion systems. *J. Chem. Phys.* 130, 094104 (doi:10.1063/1.3074302) 19275393 10.1063/1.3074302 PMC2671688

[22] Drawert B, Lawson MJ, Petzold L, Khammash M 2010 The diffusive finite state projection algorithm for efficient simulation of the stochastic reaction-diffusion master equation. *J. Chem. Phys.* 132, 074101 (doi:10.1063/1.3310809) 20170209 10.1063/1.3310809 PMC2905448

[23] Ferm L, Hellander A, Lötstedt P 2010 An adaptive algorithm for simulation of stochastic reaction–diffusion processes. *J. Comput. Phys.* 229, 343–360. (doi:10.1016/j.jcp.2009.09.030)

[24] Kalantzis G 2009 Hybrid stochastic simulations of intracellular reaction–diffusion systems. *Comput. Biol. Chem.* 33, 205–215. (doi:10.1016/j.compbiolchem.2009.03.002) 19414282 10.1016/j.compbiolchem.2009.03.002 PMC2693469

[25] Chiam KH, Tan CM, Bhargava V, Rajagopal G 2006 Hybrid simulations of stochastic reaction-diffusion processes for modeling intracellular signaling pathways. *Phys. Rev. E Stat. Nonlinear Soft Matter Phys.* 74, 1–13. (doi:10.1103/PhysRevE.74.051910) 10.1103/PhysRevE.74.051910 17279942

[26] Franz B, Flegg MB, Chapman SJ, Erban R 2013 Multiscale reaction-diffusion algorithms: PDE-assisted Brownian dynamics. *SIAM J. Appl. Math.* 70, 1224–1247. (doi:10.1137/120882469)

[27] Kalantzis G 2009 Hybrid stochastic simulations of intracellular reaction-diffusion systems. *Comput. Biol. Chem.* 33, 205–215. (doi:10.1016/j.compbiolchem.2009.03.002) 19414282 10.1016/j.compbiolchem.2009.03.002 PMC2693469

[28] Rossinelli D, Bayati B, Koumoutsakos P 2008 Accelerated stochastic and hybrid methods for spatial simulations of reaction-diffusion systems. *Chem. Phys. Lett.* 451, 136–140. (doi:10.1016/j.cplett.2007.11.055)

[29] Van Kampen NG 1992 *Stochastic processes in physics and chemistry*, vol. 1 Amsterdam, The Netherlands: Elsevier.

[30] Othmer H, Scriven L 1971 Instability and dynamic pattern in cellular networks. *J. Theor. Biol.* 32, 507–537. (doi:10.1016/0022-5193(71)90154-8) 5571122 10.1016/0022-5193(71)90154-8

[31] Bollenbach T, Pantazis P, Kicheva A, Bokel C, Gonzalez-Gaitan M, Jülicher F 2008 Precision of the Dpp gradient. *Development* 135, 1137–1146. (doi:10.1242/dev.012062) 18296653 10.1242/dev.012062

[32] Lo WC, Zhou S, Wan FYM, Lander AD, Nie Q 2014 Robust and precise morphogen-mediated patterning: trade-offs, constraints and mechanisms. *J. R. Soc. Interface* 12, 20141041 (doi:10.1098/rsif.2014.1041) 25551154 10.1098/rsif.2014.1041 PMC4277094

[33] Lander AD, Lo WC, Nie Q, Wan FYM 2009 The measure of success: constraints, objectives, and tradeoffs in morphogen-mediated patterning. *Cold Spring Harb. Perspect. Biol.* 1, a002022 (doi:10.1101/cshperspect.a002022) 20066078 10.1101/cshperspect.a002022 PMC2742077

[34] Ben-Zvi D, Barkai N 2010 Scaling of morphogen gradients by an expansion-repression integral feedback control. *Proc. Natl Acad. Sci. USA* 107, 6924–6929. (doi:10.1073/pnas.0912734107) 20356830 10.1073/pnas.0912734107 PMC2872437

[35] Lei J, Wang D, Song Y, Nie Q, Wan FYM 2012 Robustness of morphogen gradients with ‘bucket brigade’ transport through membrane-associated non-receptors. *Discrete Continuous Dyn. Syst. B* 18, 721–739. (doi:10.3934/dcdsb.2013.18.721) 10.3934/dcdsb.2013.18.721 PMC3826288 24244111

[36] Lou Y, Nie Q, Wan FYM 2005 Effects of Sog on Dpp-receptor binding. *SIAM J. Appl. Math.* 65, 1748–1771. (doi:10.1137/S0036139903433219) 17377624 10.1137/S0036139903433219 PMC1829206

[37] Koch AJ, Meinhardt H 1994 Biological pattern formation: from basic mechanisms to complex structures. *Rev. Mod. Phys.* 66, 1481–1507. (doi:10.1103/RevModPhys.66.1481)

[38] Drawert B, Engblom S, Hellander A 2012 URDME: a modular framework for stochastic simulation of reaction-transport processes in complex geometries. *BMC Syst. Biol.* 6, 76 (doi:10.1186/1752-0509-6-76) 22727185 10.1186/1752-0509-6-76 PMC3439286

